# Hydration Thermodynamics of Non-Polar Aromatic Hydrocarbons: Comparison of Implicit and Explicit Solvation Models

**DOI:** 10.3390/molecules23112927

**Published:** 2018-11-09

**Authors:** Hankyul Lee, Hyung-Kyu Lim, Hyungjun Kim

**Affiliations:** 1 Department of Chemistry, Korea Advanced Institute of Science and Technology (KAIST), Yuseong-gu, Daejeon 34141, Korea; klogw@kaist.ac.kr; 2 Division of Chemical Engineering and Bioengineering, Kangwon National University, Chuncheon, Gangwon-do 24341, Korea

**Keywords:** hydration, non-polar solute, implicit solvation, π-water hydrogen bonding, coupled QM/MM

## Abstract

The precise description of solute-water interactions is essential to understand the chemo-physical nature in hydration processes. Such a hydration thermodynamics for various solutes has been explored by means of explicit or implicit solvation methods. Using the Poisson-Boltzmann solvation model, the implicit models are well designed to reasonably predict the hydration free energies of polar solutes. The implicit model, however, is known to have shortcomings in estimating those for non-polar aromatic compounds. To investigate a cause of error, we employed a novel systematic framework of quantum-mechanical/molecular-mechanical (QM/MM) coupling protocol in explicit solvation manner, termed DFT-CES, based on the grid-based mean-field treatment. With the aid of DFT-CES, we delved into multiple energy parts, thereby comparing DFT-CES and PB models component-by-component. By applying the modified PB model to estimate the hydration free energies of non-polar solutes, we find a possibility to improve the predictability of PB models. We expect that this study could shed light on providing an accurate route to study the hydration thermodynamics for various solute compounds.

## 1. Introduction

Hydration is a key phenomenon, which governs the chemo-physical processes occurring in an aqueous solution [1]. By means of non-covalent interactions among solutes and solvents, hydration largely affects the thermodynamics of the various processes in water and, thus, is often critical in determining the energetics of chemical reaction [2], bio-molecular association [3,4], structural stabilization of protein and nucleic acids [4,5,6,7,8] and self-assembly [9]. Therefore, an in-depth understanding and an accurate quantification of the hydration free energy have been a long-standing goal of many theoretical chemists to date.

According to Ben-Naim’s definition, hydration free energy is the free-energy change associated with a transfer of an isolated solute in gas-phase into water [10,11,12]. To evaluate $\Delta G_{\mathrm{hyd}}$, a number of theoretical treatments have been developed [13,14,15,16,17,18,19,20,21,22]. These so-called solvation models treat the molecular level of details on solvents, either in an explicit or in an implicit manner. When it comes to implicit (or continuum) solvation model, in particular, various methods have been widely used to predict solvation free energies over the decades, including Poisson-Boltzmann (PB) model [13], polarizable continuum model (PCM) [14] and its variations (dielectric PCM (D-PCM) [15], conductor PCM (C-PCM) [16], and integral equation formalism (IEF-PCM) [17]), conductor-like screening model (COSMO) [18] and COSMO for real solvents (COSMO-RS) [19], generalized Born (GB) model [20], SM*x* series, and the solvent model density (SMD) model [21,22].

On the basis of an appropriate description of non-covalent interactions among solutes and solvents, explicit solvation models usually demonstrate good agreement with experimental data for small molecules of solutes [23,24,25]. However, explicit solvation models are computationally expensive, and also often use the classical molecular dynamics (MD) simulations where electronic polarization effect cannot be fully accounted for. In an implicit solvation model, on the other hand, the solvent is simply treated as a continuous dielectric medium, which enables a drastic decrease in computational cost while retaining a reasonable description of hydration free energy. However, the lack of molecular interactions limits their accuracy level, particularly in describing the hydrophobic effect or hydrogen bonds.

When non-polar solutes are dissolved in water, they tend to aggregate together, often referred to as the hydrophobic effect [26,27,28,29,30]. If one considers an aggregation process of two non-polar solutes, the difference of hydration free energies between the two monomers (say, S + S) and the aggregated dimer (S⋯S) becomes the critical thermodynamic driving force for hydrophobic aggregation:

(1)  S (aq)+S (aq)→S⋯S(aq) 

It is, thus, necessary to understand the hydration thermodynamics of hydrophobic solutes. Lum-Chandler-Weeks (LCW) theory provides a unified picture of length-scale-dependent hydration thermodynamics of non-polar solutes [31]. The theory suggests a transition from entropy-driven to enthalpy-driven hydration thermodynamic while increasing the solute size [26]. In most theoretical models, the hydrophobic solutes are often conceived as having a repulsive solute-solvent interaction, and thus have been usually modeled using hard-sphere particles. Interestingly, aromatic hydrocarbons yield the negative values of hydration free energies, in contrast to other hydrophobic solutes such as aliphatic hydrocarbons (e.g., the hydration free energy of methane is +1.91 kcal/mol while that of benzene is −0.87 kcal/mol [32]). This implies the existence of attractive solute-solvent interaction, which delicately manifests the hydration energetics of the hydrophobic aromatic molecules. Indeed, implicit solvation methods usually fail to predict the hydration free energies of aromatic molecules. For instance, PB model underestimates the value by around half and SMD models constantly underestimates the values (Figure S1).

In this article, we investigate hydration free energies of various non-polar aromatic hydrocarbons. For accurate evaluation of their hydration free energies, we employ the mean-field quantum mechanics/molecular mechanics (QM/MM) simulation, namely DFT-CES, recently developed by our group. By means of combining DFT-CES and the two-phase thermodynamic (2PT) model, we compute the hydration free energies for a set of non-polar solutes, in comparison with those obtained from an implicit solvation model. We then decompose the hydration free energy into four distinct components: reorganization, electrostatic, cavitation, and dispersion energies.

## 2. Materials and Methods

### 2.1. Density Functional Theory in Classical Explicit Solvents (DFT-CES)

In the mean-field QM/MM method [33,34,35], a QM subsystem is embedded in the mean electrostatic potential arising from MM particles, $\langle V_{\mathrm{MM}}\rangle$. Instead of numerically solving equations of the motions of QM and MM particles at the same discretized time domain, the mean-field QM/MM employs an iterative procedure consisting of QM optimizations and classical molecular dynamics (MD) simulations. At each step of the QM optimization, both the electron density ($\rho_{\mathrm{QM}}$) and the atomic structure ($r_{\mathrm{QM}}$) are subject to be minimized under the influence of $\langle V_{\mathrm{MM}}\rangle$. Additionally, at each step of the MD simulation, the MM particles at $r_{\mathrm{MM}}$, with momenta of $p_{\mathrm{MM}}$, undergo classical dynamics under the influence of van der Waals (vdW) and electrostatic potentials due to the fixed QM particles. This iterative procedure can be seen as a QM/MM version of a self-consistent reaction field (SCRF) theory to treat solvation effect, and thus can be referred to as a self-consistent ensemble-averaged reaction field (SCERF). During SCERF, if one treats QM region using Kohn-Sham density functional theory (KS-DFT), the free-energy functional of the total QM/MM system defined in Equation (2) is (approximately) minimized upon QM electronic and nuclei degrees of freedom [36]:

(2) $$A_{\mathrm{tot}}=E^{\mathrm{KS}}\left[\rho_{\mathrm{QM}};r_{\mathrm{QM}}\right]-k_{B}T\ln\int e^{-\beta H_{\mathrm{MM}}}dr_{\mathrm{MM}}^{3N}dp_{\mathrm{MM}}^{3N}.$$

On the right-hand side of Equation (2), the first term $E^{\mathrm{KS}}$, which is the KS-DFT total energy functional, denotes the internal electronic energy ($E_{\mathrm{QM}}^{\mathrm{int}}$) of the QM subsystem. $E_{\mathrm{QM}}^{\mathrm{int}}$ can be evaluated in the gas phase, although the $\rho_{\mathrm{QM}}$ and $r_{\mathrm{QM}}$ are optimized under the effects of $\langle V_{\mathrm{MM}}\rangle$. The second term of Equation (2) denotes the free energy ($A_{\mathrm{MM}}$) of the MM subsystem, where $H_{\mathrm{MM}}$ is the classical Hamiltonian governing the MM particle dynamics under an external potential describing the QM subsystem.

Recently, our group developed a grid-based mean-field QM/MM method, which incorporates the periodic DFT with plane-wave basis set, coupled with the classical MD. For brevity, the QM/MM method is called *DFT in Classical Explicit Solvents*, namely *DFT-CES* [37]. DFT-CES is designed to utilize a very fine three-dimensional rectangular grid as a communication medium between QM and MM subsystems, thereby transferring precise information of $\rho_{\mathrm{QM}}$ and $\langle V_{\mathrm{MM}}\rangle$ seamlessly to the MM and QM subsystem, respectively, without involving a charge fitting scheme, such as an electrostatic potential (ESP) fitting procedure [38]. In addition, by performing both DFT and MD simulations in the periodic box, long-range electrostatic interaction is straightforwardly taken into consideration when solving the Poisson equation by using the fast Fourier transform method.

Detail of the DFT-CES method is fully provided in our recent literature [37].

### 2.2. Free-Energy Calculation Using Two-Phase Thermodynamic (2PT) Model

To compute the hydration free energy ($\Delta A_{\mathrm{hyd}}$) using DFT-CES, a natural partitioning of the total hydrated system is the QM description on the solute and MM description on the water. After the SCERF iterations, the converged DFT results of $\rho_{\mathrm{QM}}$ and $r_{\mathrm{QM}}$ enable us to evaluate the electronic and structural reorganization energy of the QM solute, which is $E_{\mathrm{reorg}}=E_{\mathrm{QM}\left(\mathrm{solute}\right)}^{\mathrm{int}}-E_{\mathrm{QM}\left(\mathrm{solute}\right)}^{0}$. Here, $E_{\mathrm{QM}\left(\mathrm{solute}\right)}^{\mathrm{int}}$ (or $E_{\mathrm{QM}\left(\mathrm{solute}\right)}^{0}$) is the KS-DFT total energy of the QM solutes in the gas phase, fully optimized in the presence (or in the absence) of $\langle V_{\mathrm{MM}}\rangle$.

Having the converged $\rho_{\mathrm{QM}}$ and $r_{\mathrm{QM}}$ of the hydrated QM solute after the SCERF iteration, the classical dynamics of MM water subsystem under the fixed solute potential is well defined, and the free energy of the MM water subsystem, $A_{\mathrm{MM}\left(\mathrm{wat}\right)}$ is given by the second term of Equation (2). By defining $A_{\mathrm{MM}\left(\mathrm{wat}\right)}^{0}$ as the free energy of the bulk water (i.e., a reference state), the free energy change of water associated with the hydration process becomes $\Delta A_{\mathrm{wat}}=A_{\mathrm{MM}\left(\mathrm{wat}\right)}-A_{\mathrm{MM}\left(\mathrm{wat}\right)}^{0}$, and the hydration free energy is defined as:

(3) $$\;\Delta A_{\mathrm{hyd}}=E_{\mathrm{reorg}}+\Delta A_{\mathrm{wat}}\;$$

The free-energy change of $\Delta A_{\mathrm{wat}}$ can be calculated by means of an alchemical transformation such as free-energy perturbation [39] or thermodynamic integration [40,41,42]. During MD simulations of the water subsystem, such a calculation is commonly achieved by switching on and off the fixed solute potential. To speed up the evaluation of $A_{\mathrm{MM}\left(\mathrm{wat}\right)}$ and $A_{\mathrm{MM}\left(\mathrm{wat}\right)}^{0}$, instead, we choose the two-phase thermodynamic (2PT) model to extract directly thermodynamic properties from MD trajectories [43,44,45,46]. For a given liquid system, the 2PT model decomposes the total number of its degrees of freedom into two parts (gas-like and the solid-like parts), as inheriting the viewpoint of Eyring’s significant theory of liquids [47,48]. Then, the free energy of the liquid system is assumed to be given as the linear combination of the free energy of the gas-like part and that of the solid-like part. The thermodynamics of the gas-like part is analytically calculated by considering a hard-sphere system having the corresponding effective packing fraction, and the thermodynamics of the solid-like part is numerically calculated using harmonic oscillator model.

The 2PT model has been widely tested and proven successful for the quantitative prediction of the thermodynamics and phase behavior of Lennard-Jones particles at various densities [43], absolute entropy of water molecules [44] and organic solvents [45], and the interfacial thermodynamics of various liquids, e.g., surface tensions [46]. It is further emphasized that the combination of DFT-CES and 2PT has demonstrated a satisfactory description of the $\Delta A_{\mathrm{hyd}}$ of the 17 polar solutes [37], which were adopted as the official SAMPL0 challenge test set [25], and of the solid-liquid interfacial tension of water-graphene/graphite system [49].

### 2.3. Simulation Details

Using our in-house code, we implemented DFT-CES by coupling two open-source programs of Quantum Espresso (plane-wave DFT engine) [50] and the LAMMPS (classical MD engine) [51]. For the DFT part, we employed the Perdew-Burke-Ernzerhof (PBE) exchange-correlation functional [52] with the projector augmented-wave (PAW) scheme [53,54], and set the kinetic energy cutoff as 50 Ry. For the MD part, we performed the canonical ensemble (NVT) simulations at 300 K, using the Nosé-Hoover thermostat [55,56] with a damping constant of 100 fs. During the MD part of each SCERF iteration, we performed a total of 2.5 ns NVT MD sampling, where the last 2 ns of the trajectory was employed to evaluate $\langle V_{\mathrm{MM}}\rangle$. Solute-water vdW interactions were described using the OPLS-AA force field [57]. Intermolecular interactions of waters were described using the modified TIP3P water potential [58], wherein all bond lengths and angles were constrained to their equilibrium value with the RATTLE algorithm [59]. During MD, long-range Coulomb interactions were treated using the Ewald summation method [60] with a real space cut-off of 15 Å.

**Figure 1:**
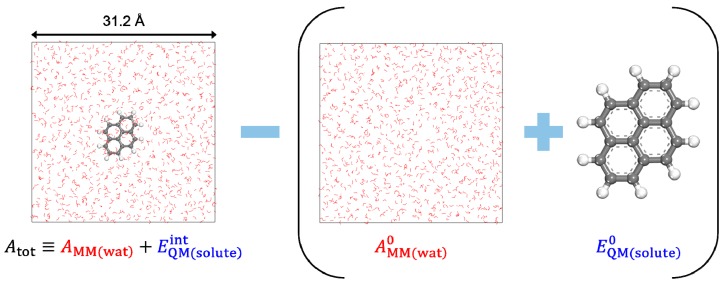
Schematized process of calculating the hydration free energy ($\Delta A_{\mathrm{hyd}}$). In the perspective of the hydration process, the first term corresponds to the water-solvated system ($A_{\mathrm{MM}\left(\mathrm{wat}\right)}+E_{\mathrm{QM}\left(\mathrm{solute}\right)}^{\mathrm{int}}$), whereas the second term (in parentheses) corresponds to the reference states for the bulk water box ($A_{\mathrm{MM}\left(\mathrm{wat}\right)}^{0}$) and the QM solute in the gas-phase ($E_{\mathrm{QM}\left(\mathrm{solute}\right)}^{0}$).

DFT-CES simulation box contains a QM solute at the center, which is hydrated by 1,000 classical water molecules. To compute the energies of reference states, $E_{\mathrm{QM}\left(\mathrm{solute}\right)}^{0}$ and $A_{\mathrm{MM}\left(\mathrm{wat}\right)}^{0}$, we separately performed a DFT calculation for the gas-phase optimization of the QM solute and an MD simulation of the bulk water box, respectively. Figure 1 illustrates the simulation box for the representative case. The SCERF iteration of the DFT-CES calculation was repeated until the convergence criterion was sufficiently achieved. The criterion is set such that the DFT energy difference becomes less than 0.1 kcal/mol.

## 3. Results and Discussion

### 3.1. Hydration Free Energies of Aromatic Hydrocarbons

**Figure 2:**
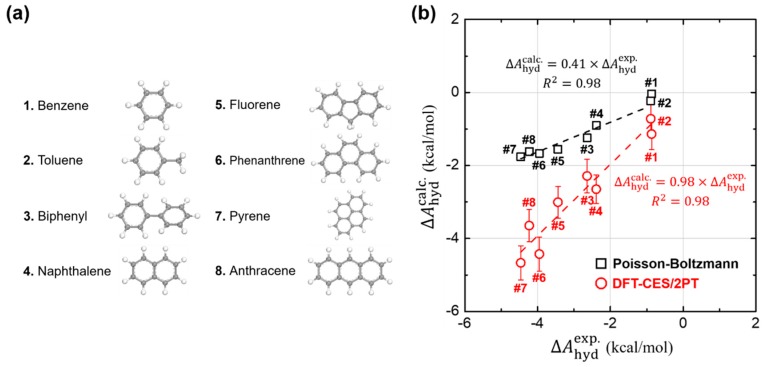
Calculation of hydration free energies ($\Delta A_{\mathrm{hyd}}^{\mathrm{calc}.}$) of (**a**) eight non-polar aromatic hydrocarbons. (**b**) Comparison of our DFT-CES/2PT (red circles) and the Poisson-Boltzmann (PB) implicit solvation model (black squares) with respect to the experimental $\Delta A_{\mathrm{hyd}}^{\exp.}$ results. Our DFT-CES/2PT method gives an excellent estimate of the experimental $\Delta A_{\mathrm{hyd}}^{\exp.}$ results. The $\Delta A_{\mathrm{hyd}}^{\mathrm{calc}.}$ values calculated using the PB model, however, are underestimated by ca. 40% of the experimental ones. $R^{2}$ is the coefficient of determination, as calculated by linear least-squares analysis.

Using DFT-CES/2PT method, we calculated $\Delta A_{\mathrm{hyd}}$ of eight non-polar aromatic hydrocarbons, listed as benzene, toluene, biphenyl, naphthalene, fluorene, phenanthrene, pyrene, and anthracene (Figure 2a). For comparison, we also evaluated $\Delta A_{\mathrm{hyd}}$ by applying the Poisson-Boltzmann (PB) implicit solvation model as implemented in Jaguar 8.4 [61]. Such DFT calculations were carried out with the choice of Perdew-Burke-Ernzerhof (PBE) exchange-correlation functional [52] and 6–31G ** basis sets [62,63]. We note that our test set of eight molecules, likewise as most of the other aromatic hydrocarbons, do not exhibit much flexibility in their conformations so that a rigorous conformational sampling analysis is not significant. In Figure 2b, the calculated $\Delta A_{\mathrm{hyd}}$ values are compared with the available experimental results [64,65]. We find that our DFT-CES/2PT method shows a remarkable performance in predicting the $\Delta A_{\mathrm{hyd}}$ values of aromatic hydrocarbons, which is characterized by small mean absolute error (MAE) and root-mean-square error (RMSE) of 0.34 and 0.37 kcal/mol, respectively. The implicit PB solvation model, however, shows a significant underestimating tendency, estimating only ca. 40% of experimental $\Delta A_{\mathrm{hyd}}$ results. Considering that the PB model in Jaguar is well optimized to accurately predict the $\Delta A_{\mathrm{hyd}}$ values for polar solutes (MAE = 1.73 kcal/mol and RMSE = 1.88 kcal/mol for the SAMPL0 test set; Figure 2), such a gross error in predicting the aromatic hydrocarbons is notable.

To trace the error source of the PB solvation model, we perform a comparative study between DFT-CES and PB calculation results. Implicit solvation models, including the PB solvation model, generally assume that the total hydration free energy ($\Delta A_{\mathrm{hyd}}$) is given as the sum of the electronic/structural reorganization energy of the solute $\left(E_{\mathrm{reorg}}\right)$, and electrostatic and non-electrostatic free energies (which are respectively denoted as $\Delta A_{\mathrm{ES}}$ and $\Delta A_{\mathrm{nonES}}$), where $\Delta A_{\mathrm{nonES}}$ consists of the cavitation ($\Delta A_{\mathrm{cav}}$) and dispersion ($\Delta A_{\mathrm{disp}}$) components:

(4) $$\;\Delta A_{\mathrm{hyd}}=E_{\mathrm{reorg}}\;+\;\Delta A_{\mathrm{ES}}\;+\Delta A_{\mathrm{nonES}}\;$$

(5) $$\;\Delta A_{\mathrm{nonES}}=\Delta A_{\mathrm{cav}}\;+\;\Delta A_{\mathrm{disp}}\;$$

We now decompose the estimate of $\Delta A_{\mathrm{hyd}}$ from the DFT-CES into four different contributions: $E_{\mathrm{reorg}}$, $\Delta A_{\mathrm{ES}}$, $\Delta A_{\mathrm{cav}}$, and $\Delta A_{disp}$. The component of $E_{\mathrm{reorg}}$ can be readily separated from $\Delta A_{\mathrm{hyd}}$, as being defined as the first term of Equation (3). To extract the rest of the term ($\Delta A_{\mathrm{wat}}$), we additionally carry out a series of DFT-CES/2PT calculations in a non-self-consistent manner without involving SCERF iteration. $\Delta A_{\mathrm{ES}}$ is first decomposed from $\Delta A_{\mathrm{wat}}$, with the help of the non-self-consistent DFT-CES/2PT simulation where the electrostatic potential of the QM solute (which is fully stored in the grid) is switched off. $\Delta A_{\mathrm{disp}}$ is further decomposed using the non-self-consistent DFT-CES/2PT result where the vdW potential is replaced with the purely repulsive Weeks-Chandler-Anderson (WCA) potential [66]. The remaining term, which is associated with the switching-off process of the WCA potential, becomes $\Delta A_{\mathrm{cav}}$. (For more information, please refer to Equations (15)–(17) in [37].) Each energy component decomposed from $\Delta A_{\mathrm{hyd}}$ is summarized in Table 1.

### 3.2. Validity of Linear-Reponse Theory

In the implicit solvation models, linear-response theory (LRT) is underlying to estimate the “free energy quantity of ΔA” from the easily computable “interaction energy of U (having no entropic term)”. Considering the reversible work done by water during the process of turning on solute-solvent interaction from $U_{\mathrm{uv}}$ to $U_{\mathrm{uv},0}$, the LRT yields $\Delta A_{\mathrm{wat}}=\left(U_{\mathrm{uv}}+U_{\mathrm{uv},0}\right)/2$ [67]. For the process of turning the electrostatic interaction on, the solvent medium is expected to be gradually polarized by re-orienting the dipoles of water molecules. Thus, the electrostatic interaction energy is assumed to be grown from $U_{\mathrm{ES},0}=0$ to a finite value of $U_{\mathrm{ES}}$, resulting in $\Delta A_{\mathrm{ES}}=U_{\mathrm{ES}}/2$.

**Figure 3:**
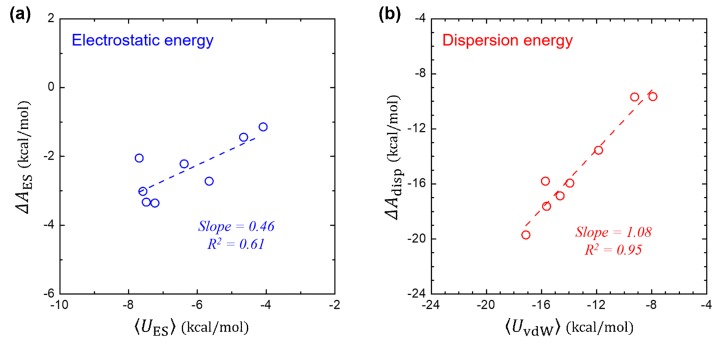
Validation test of linear-response theory. (**a**) Comparison between the electrostatic component ($\Delta A_{\mathrm{ES}}$) in $\Delta A_{\mathrm{hyd}}$ and the ensemble-average of the solute-solvent electrostatic interaction energy $\langle U_{ES}\rangle$. (**b**) Comparison between the dispersion component ($\Delta A_{\mathrm{disp}}$) of $\Delta A_{\mathrm{hyd}}$ and the ensemble-average of the solute-solvent vdW interaction energy $\langle U_{\mathrm{vdW}}\rangle$.

In Figure 3a, we compare the free-energy component of $\Delta A_{\mathrm{ES}}$ (that is computed using 2PT method) and its corresponding interaction energy of $\langle U_{ES}\rangle$ (that is ensemble averaged over the MD trajectories). It shows a linear relationship of $\Delta A_{\mathrm{ES}}\;\approx \;0.46\langle U_{\mathrm{ES}}\rangle$, where the slope of 0.46 is in consistent not only with the theoretical value from the LRT (slope = 0.5), but also with the value (slope ≈ 0.40) determined for the polar solutes in the SAMPL0 set [37]. This implies that the LRT can be applied for both polar and non-polar solutes with a reasonable accuracy. The global linear fit on the polar and non-polar solutes leads to the linear coefficient of 0.41 with $R^{2}=0.95$.

While gradually tuning the dispersive vdW interaction on, no dipolar relaxation of the water is expected, yielding $U_{\mathrm{vdW},0}=U_{\mathrm{vdW}}$ and thereby $\Delta A_{\mathrm{disp}}=U_{\mathrm{vdW}}$. Figure 3b further shows the near identity between the $\Delta A_{\mathrm{disp}}$ and the ensemble-averaged $\langle U_{vdW}\rangle$, in consistent with the LRT about vdW interaction.

### 3.3. Non-Electrostatic Interaction Components: Surface-Area Dependence

The PB solvation model evaluates $\Delta A_{\mathrm{ES}}$ based on the LRT by calculating the electrostatic interaction ($U_{\mathrm{ES}}$) between the solute and the SCRF that is obtained by solving a continuum equation such as a Poisson-Boltzmann equation. However, $\Delta A_{\mathrm{disp}}$ cannot be obtained using the LRT due to the difficulty in calculating $U_{\mathrm{vdW}}$, but is estimated by simply assuming its linear relationship with the solvent accessible surface area (ASA) in the PB solvation model. Additionally, the cavitation free energy of $\Delta A_{\mathrm{cav}}$ is conceived to be proportional to either the surface area or the volume of the solute, which is rationalized using the scaled-particle theory for hard spheres [68].

**Figure 4:**
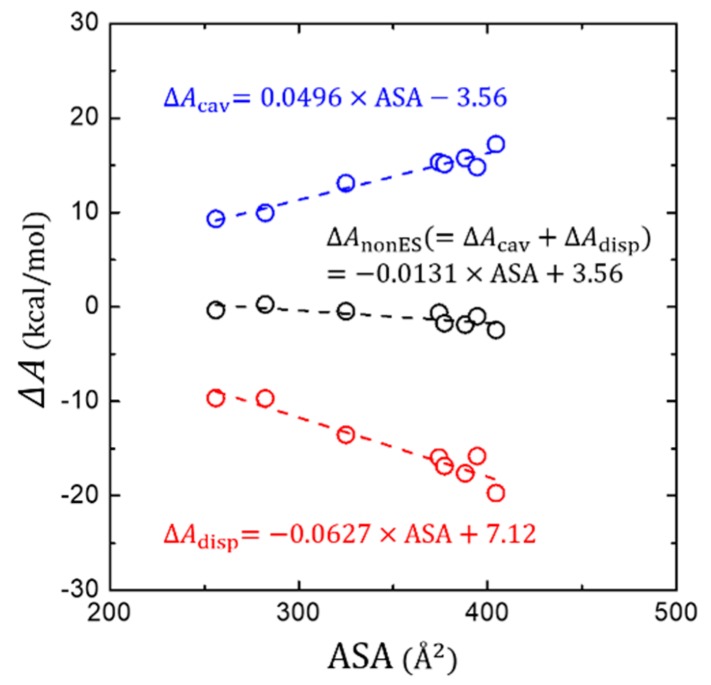
Change of the non-electrostatic interaction components ($\Delta A_{\mathrm{cav}}$, $\Delta A_{\mathrm{disp}}$, and their total sum $\Delta A_{\mathrm{nonES}}$) in terms of the solvent-accessible surface area (ASA).

Figure 4 shows how $\Delta A_{\mathrm{disp}}$ or $\Delta A_{\mathrm{cav}}$ changes as a function of ASA of the solute molecules. We find a good positive proportionality of $\Delta A_{\mathrm{disp}}$ and a negative proportionality of $\Delta A_{\mathrm{cav}}$ with the ASA, resulting in a mutual cancellation when both terms are combined into the $\Delta A_{\mathrm{nonES}}$. At a glance, these trends are generally as similar as for polar solutes [37]. However, the degree of mutual cancellation is somewhat different. The more effective stabilization of $\Delta A_{\mathrm{disp}}$ is found for the larger aromatic hydrocarbon solutes, yielding a small, but still appreciable negative proportionality of $\Delta A_{\mathrm{nonES}}$ with the ASA. This is in contrast to the case of polar solutes, where a more complete mutual cancellation has been found [37].

### 3.4. Component-by-Component Comparisons

**Figure 5:**
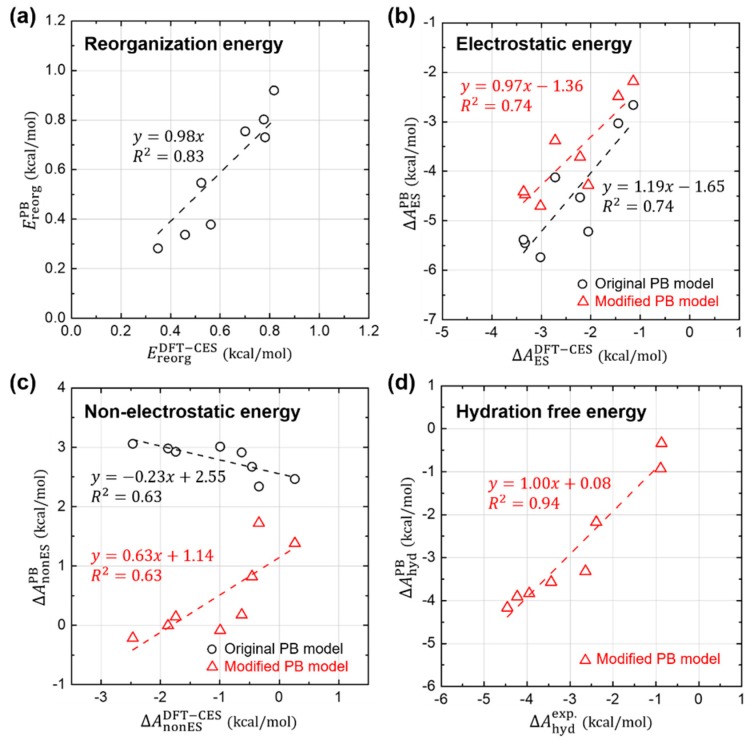
Quantitative comparison between DFT-CES/2PT and PB calculation results. (**a**) Reorganization, (**b**) Electrostatic, (**c**) Non-electrostatic, and (**d**) their total hydration free energies, obtained from the original PB model (black circles). Values obtained from the possible modification of each term is also displayed (red triangle).

In the previous sections, we confirm that the basic approximations underlying the PB solvation model are more-or-less reasonably working still for aromatic hydrocarbons; (1) the LRT is still valid, and (2) both $\Delta A_{\mathrm{disp}}$ and $\Delta A_{\mathrm{cav}}$ are directly proportional to the ASA. In this section, we further analyze each component of $\Delta A_{\mathrm{hyd}}$ in detail, for the sake of quantitative comparison between DFT-CES and PB calculations, as shown in Figure 5.

Figure 5a shows that the PB model predicts quite well the reorganization energies ($E_{\mathrm{reorg}}$) and their absolute values are relatively small with a weak influence on the total solvation free energy.

For the case of the electrostatic component ($\Delta A_{\mathrm{ES}}$), Figure 5b shows that the PB model predicts reasonably well the relative difference of $\Delta A_{\mathrm{ES}}$. When the proportional coefficient of the LRT is changed from 0.5 to 0.41 as determined from the Section 3.2, the relative difference of $\Delta A_{\mathrm{ES}}$ is even more reliably estimated using the PB model, as shown in the linear-fit slope on the PB versus DFT-CES values curve changing from 1.19 to 0.97.

**Figure 6:**
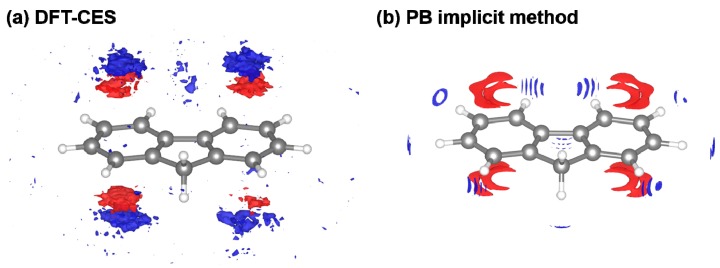
Visualized reaction-field map from (**a**) DFT-CES method and (**b**) PB implicit method. (red: positive, blue: negative charge) The map shows the ensemble-averaged solvent charge density of the solute of fluorine, as a representative case. Our DFT-CES method can successfully capture the benzene-water hydrogen bonding characters, originated from the quadrupole-dipole interactions.

However, $\Delta A_{\mathrm{ES}}$ from the PB model tends to be overestimated as a whole with a 1.65 or 1.36 kcal/mol offset when using the original and modified LRT coefficient, respectively. We attribute the offset error to the incomplete description on the reaction field. It is worth to note that aromatic hydrocarbons are known to develop π-water hydrogen bonds based on the substantial quadrupole-dipole interaction. Figure 6 shows the reaction-field maps for fluorine, as a representative example. (The other maps for the remaining seven non-polar solutes are available in Figure S2). By using the explicit solvation model of DFT-CES, in Figure 6a, the map successfully exhibits the local solvent-density fluctuation with positive and negative charges alternatively, compared with the map from the PB implicit method showing only one polarity of the positive charge near π-electron. Furthermore, our DFT-CES model can accurately describe the π-water hydrogen bonding interaction, which is known experimentally to be crucial in non-polar aromatic compounds.

The systematic offset in predicting $\Delta A_{\mathrm{ES}}$ is one source of error originated by the incompleteness of the PB model, which can bring an overestimating trend of $\Delta A_{\mathrm{hyd}}$ by 1 to 2 kcal/mol. However, it is notable that an underestimating, instead of overestimating, trend of $\Delta A_{\mathrm{hyd}}$ has been found (Figure 2), which implies that the electrostatic contribution is not the major error source of the $\Delta A_{\mathrm{hyd}}$ of the PB model.

Figure 5c shows the non-electrostatic component ($\Delta A_{\mathrm{nonES}}$) from the PB model, compared with that from the DFT-CES calculation. We note that the PB results show a significant error in predicting $\Delta A_{\mathrm{nonES}}$, which is even *anti*-correlated with the DFT-CES results with an offset of 2.55 kcal/mol. The PB model, implemented in Jaguar, yields $\Delta A_{\mathrm{nonES}}$ by using the linear-regression result, obtained from the linear and branched aliphatic hydrocarbons [69]. The calculated data produced from the original PB model shows a positive correlation between $\Delta A_{\mathrm{nonES}}$ (in unit of kcal/mol) and the ASA (in unit of Å^2^):

(6) $$\;\Delta A_{\mathrm{nonES}}^{\mathrm{PB}}=0.005\times \mathrm{ASA}+1.09\;$$

However, from our analysis on the DFT-CES results, as aforementioned in Section 3.3, the modified linear regression between $\Delta A_{\mathrm{nonES}}$ and the ASA yields the negative slope of −0.0131 kcal/mol/Å^2^ and the intercept of 3.56 kcal/mol.

We now apply two modifications, which are derived from our component-by-component analyses, to the PB model; (1) change of the linear coefficient of the LRT from 0.5 into 0.41 when evaluating $\Delta A_{\mathrm{ES}}$, and (2) use of the modified linear model predicting $\Delta A_{\mathrm{nonES}}$ from the ASA. Although the offset error of 1.36 kcal/mol found in the electrostatic component ($\Delta A_{\mathrm{ES}}$) propagates to $\Delta A_{\mathrm{hyd}}$, it is unfortunately deemed to be an innate limitation of the PB model ignoring the solvent shell structure. As an *ad-hoc* remedy, we here make the non-electrostatic component to cancel the offset error in the $\Delta A_{\mathrm{ES}}$. To help readers’ understanding, results without including such an ad hoc correction are also provided in Figure S3. From the linear-regression results on the DFT-CES data, we simply change the intercept value from the 3.56 kcal/mol to 4.92 (=3.56 + 1.36) kcal/mol:

(7) $$\;\Delta A_{\mathrm{nonES}}^{\mathrm{modPB}}=-0.0131\times \mathrm{ASA}+4.92\;$$

In Figure 5c, the newly predicted results of $\Delta A_{\mathrm{nonES}}^{\mathrm{modPB}}$ are also depicted (in red triangles) with respect to the DFT-CES results. The refined PB model offers much improved estimates of $\Delta A_{\mathrm{nonES}}^{\mathrm{modPB}}$, albeit not fully satisfactory, and also a positive correlation between $\Delta A_{\mathrm{nonES}}^{\mathrm{modPB}}$ and $\Delta A_{\mathrm{nonES}}^{\mathrm{DFT}-\mathrm{CES}}$.

Figure 5d shows the new estimates of $\Delta A_{\mathrm{hyd}}$ from the modified PB model, which are compared with the experimental values. Despite of some discrepancies in each component of $\Delta A_{\mathrm{hyd}}$, the refined PB values of $\Delta A_{\mathrm{hyd}}$ agree fairly well with the experimental data (MAE = 0.29 kcal/mol and RMSE = 0.36 kcal/mol).

**Figure 7:**
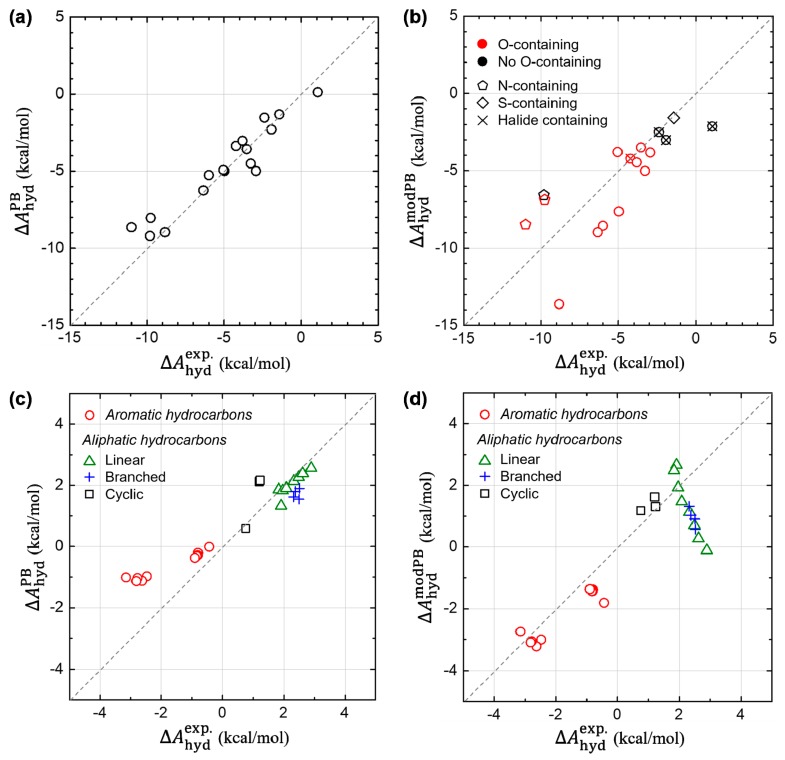
Assessment of the prediction accuracy of the original and modified PB models. The test compounds were taken from the SAMPL0 test set consisting of 17 polar solutes (in (**a**,**b**)) as well as other various types of aromatic and aliphatic hydrocarbons (in (**c**,**d**)). (Left panels (**a**,**c**)) Calculated estimates of $\Delta A_{\mathrm{hyd}}$ from the original PB model. (Right panels (**b**,**d**)) Refined estimates of $\Delta A_{\mathrm{hyd}}$ from the modified PB model.

In Figure 7, we further check the transferability of our modified PB model into other various compounds, including the 17 polar solutes adopted as the SAMPL0 test set. For the SAMPL0 test compounds, Figure 7a,b show the results from the original PB model in Jaguar and from our modified PB model, denoted as $\Delta A_{\mathrm{nonES}}^{\mathrm{PB}}$ and $\Delta A_{\mathrm{nonES}}^{\mathrm{modPB}}$, respectively. At a glance, we find that the good predictability of the original PB model becomes deteriorated, when we modify the parameters using the ones derived from non-polar aromatic hydrocarbon system. However, a deeper look on data, in Figure 7b, allows us to suggest a direction for further possible modifications of the implicit model to incorporate both polar and non-polar solutes. We find that the modified PB model can predict relatively well the estimates of $\Delta A_{\mathrm{hyd}}$, for relatively more hydrophobic solutes, such as halide-containing solutes as well as no nitrogen (N)- nor oxygen (O)-containing solutes. This infers that the newly derived parameters from the aromatic hydrocarbon could rather generally be utilized for the non-polar solutes, although more intensive tests are required. In addition, we find that the modified PB model systematically underestimates $\Delta A_{\mathrm{hyd}}$ of the N-containing solutes, while overestimates $\Delta A_{\mathrm{hyd}}$ of the O-containing solutes. This implies that it is strongly necessary to treat atom–species dependency, for the sake of improving predictability of the PB model. Considering that $\Delta A_{\mathrm{cav}}$ is estimated from a dummy-atom model (where no atom-species information is left), we suggest that the atom-species dependency needs to be included in developing a model for $\Delta A_{\mathrm{disp}}$.

Figure 7c,d also show the results from the original and modified PB models, respectively, for the other test sets including aromatic and aliphatic (linear, branched and cyclic) hydrocarbons. Concerning other ten aromatic hydrocarbons (*t*-butylbenzene, ethylbenzene, *p*-xylene, *m*-xylene, *o*-xylene, 1,3-dimethylnaphthalene, 2,6-dimethylnaphthalene, 2,3-dimethylnaphthalene, 1,4-dimethylnaphthalene, and acenaphthene), the original PB model still seems problematic as shown in Figure 7c and Figure S4 (The slope of the linear-fit curve: 0.38). On the other hand, our modified PB model also predicts well for those non-polar compounds (slope: 1.13). Moreover, we assessed the prediction accuracy of the both PB models for other aliphatic compounds. The modified PB model works reasonably well for cyclic hydrocarbons (cyclopropane, cyclopentane, and cyclohexane). However, for branched (isobutane, isopentane, neopentane, and isohexane) and linear (methane, ethane, propane, *n*-butane, *n*-pentane, *n*-hexane, *n*-heptane, and *n*-octane) hydrocarbons, Figure 7d represents a negative trend for estimating $\Delta A_{\mathrm{hyd}}$. We conceive that the origin of the gross error found in the linear and branched hydrocarbons is mostly due to the lack of conformational sampling since the ASA changes significantly during their structural dynamics. In addition, the change of conformational entropy during hydration process needs to be taken into consideration for a reliable prediction of hydration free energies of these flexible molecules, which is entirely absent in current implicit solvation methods. Indeed, it is found that the error of small molecules (e.g., methane, ethane, propane) is acceptable, while the error increases as the solute molecule becomes longer and thereby more flexible. These results further pointed out a possible problem of the previous parametrization method of the original PB model, which was carried out by comparing the experimental hydration free energy and the ASA of one conformer of the flexible linear hydrocarbon. By performing a similar comparative study of DFT-CES and PB model in our next study, we expect to build more systematic understanding on the hydration process of linear and branched hydrocarbons and then to answer the open question about the origin of the different solvation of aliphatic and aromatic hydrocarbons.

## 4. Conclusions

In this work, we examined the thermodynamic properties in the hydration process of non-polar aromatic compounds. The in-depth case study on their hydration free energies ($\Delta A_{\mathrm{hyd}}$) was carried out with the aid of a mean-field QM/MM method, i.e., DFT-CES, recently developed in our group. Here, we tested the extensibility of DFT-CES toward non-polar species, which are commonly known to be difficult challenges for general implicit solvation models. Our DFT-CES/2PT method successfully offers an excellent estimate of $\Delta A_{\mathrm{hyd}}$ for non-polar solutes, compared with the experimental dataset, whereas, for the same set of data, almost all $\Delta A_{\mathrm{hyd}}$ is poorly determined by the PB implicit model with large prediction error (ca. 40%). To delve into a cause of error, we decomposed $\Delta A_{\mathrm{hyd}}$ into multiple energy components, thereby conducting component-by-component comparisons between DFT-CES and PB models. It is noteworthy that the PB implicit model can accurately predict the experimental values in hydration free energies of aromatic hydrocarbons, after revising $\Delta A_{\mathrm{ES}}^{\mathrm{PB}}$ and $\Delta A_{\mathrm{nonES}}^{\mathrm{PB}}$ energy parts. By further testing our modified PB model against to the polar solutes, we find an atom-species dependent error, suggesting a need for a more elaborated method accounting for exposed atom-species dependent modeling of $\Delta A_{\mathrm{disp}}$. We anticipate that our study provides not only a fundamental understanding on the hydration thermodynamics of non-polar aromatic hydrocarbons, but also a route to overcome limitations of implicit solvation methods toward an improved accuracy for extended sets of solute molecules.

## Figures and Tables

**Table 1:** Component-by-component analysis for the hydration free energies ($\Delta A_{\mathrm{hyd}}$) by using DFT-CES/2PT method. $\Delta A_{\mathrm{hyd}}$ is decomposed into the reorganization energy ($E_{\mathrm{reorg}}$), electrostatic term ($\Delta A_{\mathrm{ES}}$), dispersion term ($\Delta A_{\mathrm{disp}})$, and cavitation energy ($\Delta A_{\mathrm{cav}}$). Units are in kcal/mol.

Solute Molecule	$\;\Delta A_{hyd}\;$	$\;E_{reorg}\;$	$\;\Delta A_{wat}\;$		
			$\Delta A_{ES}$	$\Delta A_{disp}$	$\Delta A_{cav}$
#1. Benzene	−1.14	+0.35	−1.14	−9.66	+9.32
#2. Toluene	−0.72	+0.46	−1.45	−9.69	+9.95
#3. Biphenyl	−2.29	+0.56	−2.22	−15.94	+15.31
#4. Naphthalene	−2.65	+0.52	−2.72	−13.56	+13.10
#5. Fluorene	−3.01	+0.78	−2.05	−16.87	+15.13
#6. Phenanthrene	−4.43	+0.78	−3.33	−17.63	+15.76
#7. Pyrene	−4.67	+0.82	−3.02	−19.70	+17.23
#8. Anthracene	−3.65	+0.70	−3.36	−15.80	+14.81

## Supplementary Materials

The supplementary materials are available online.
