# Supplementary material for: Hydration Thermodynamics of Non-Polar Aromatic Hydrocarbons: Comparison of Implicit and Explicit Solvation Models
Source: Molecules. 2018 Nov 9;23(11):2927. doi: 10.3390/molecules23112927 (PMC6278349; doi:10.3390/molecules23112927)

molecules

MDPI

Supplementary Materials

Hydration Thermodynamics of Non-Polar Aromatic Hydrocarbons: Comparison of Implicit and Explicit Solvation Models

Hankyul Lee [1], Hyung-Kyu Lim [2,*] and Hyungjun Kim [1,*]

Figure S1. Performance test of Poisson-Boltzmann (PB), SM8, and SM6 implicit solvation methods for eight non-polar aromatic hydrocarbons. All DFT calculations were carried out with Perdew-Burke-Ernzerhof (PBE) exchange-correlation functional and 6-31G** basis sets. Indeed, implicit solvation methods usually fail to predict the hydration free energies of aromatic molecules. For instance, PB model underestimates the value by around half and SMD models constantly underestimates the values.

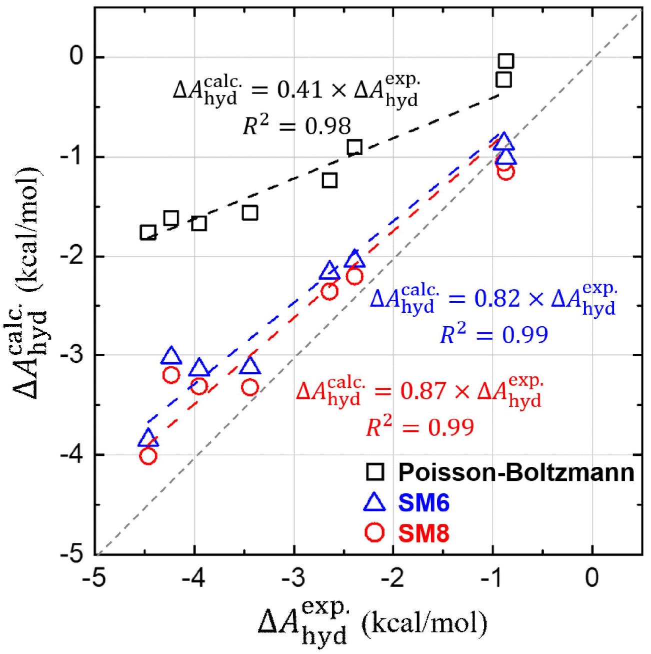

Figure S2. Visualized reaction-field map from our DFT-CES method (left) and PB implicit method (right). (red: positive, blue: negative charge) The DFT-CES method (left panel) can successfully describe the local solvent-density fluctuation with alternative +/- charges, regarding the benzene-water hydrogen bonding characters.

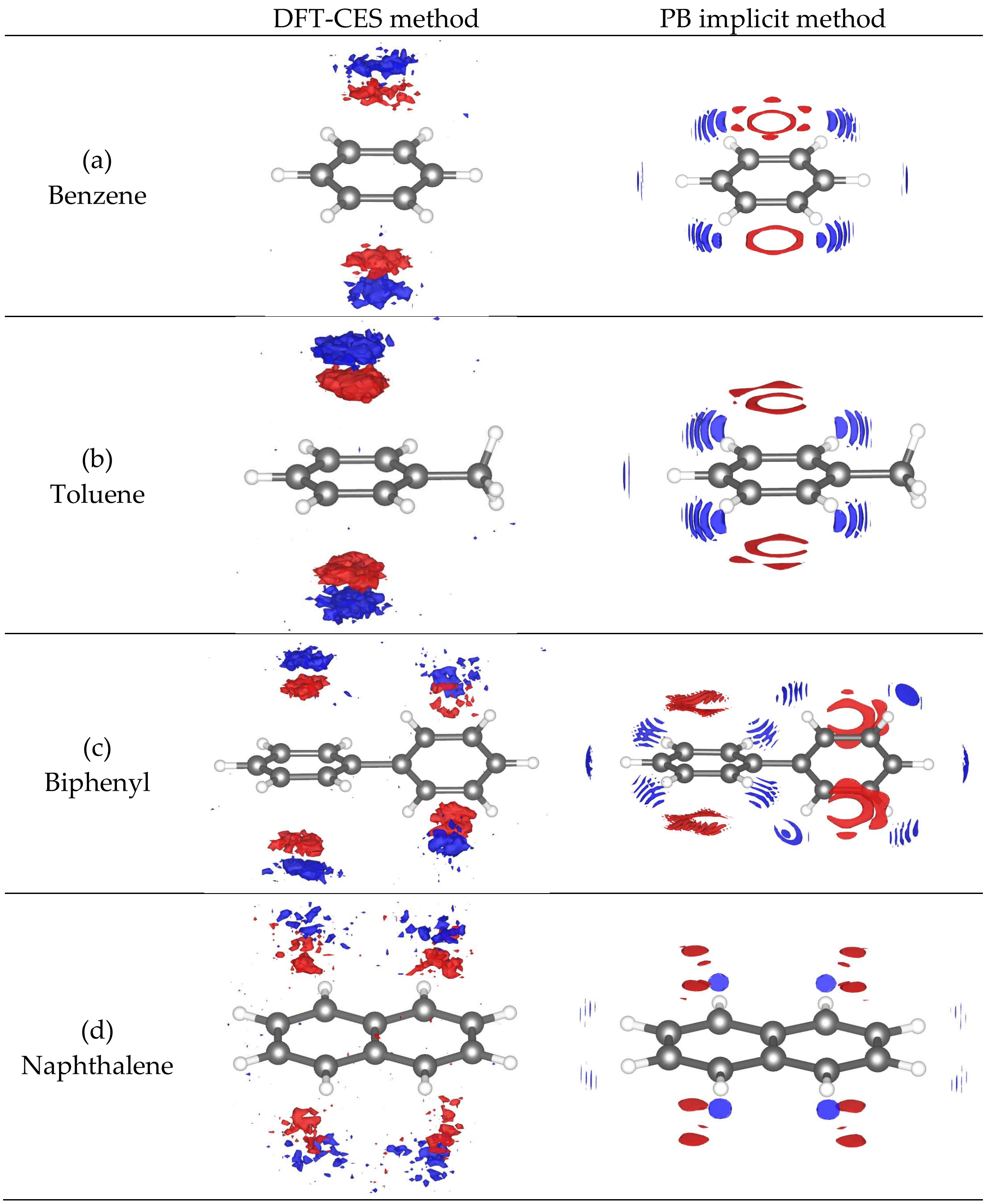

(e) Phenanthren e	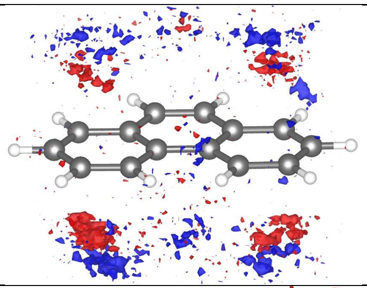	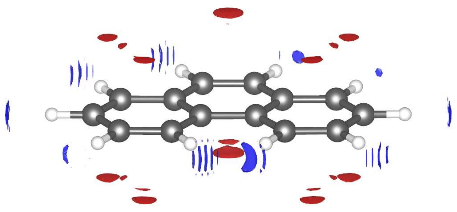
(f) Pyrene	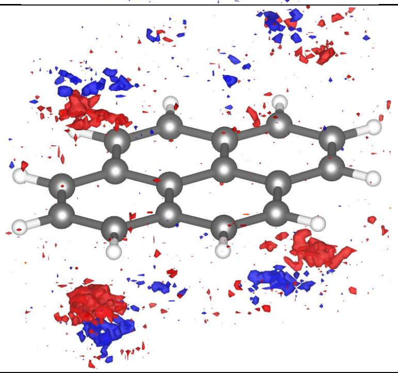	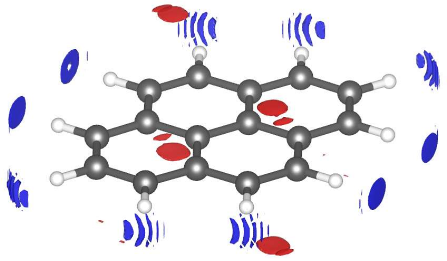
(g) Anthracene	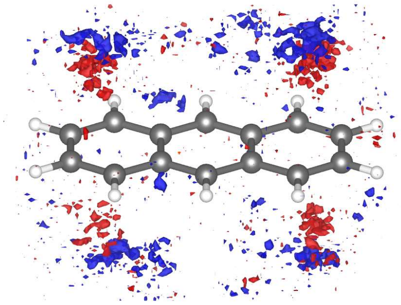	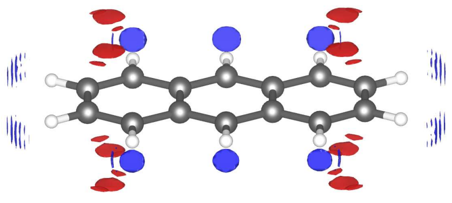

Figure S3. Influence of the *ad-hoc* offset correction in the electrostatic components. Comparison of the total hydration free energies between with and without the offset correction for (a) non-polar solutes as well as (b) 17 types of polar solutes.

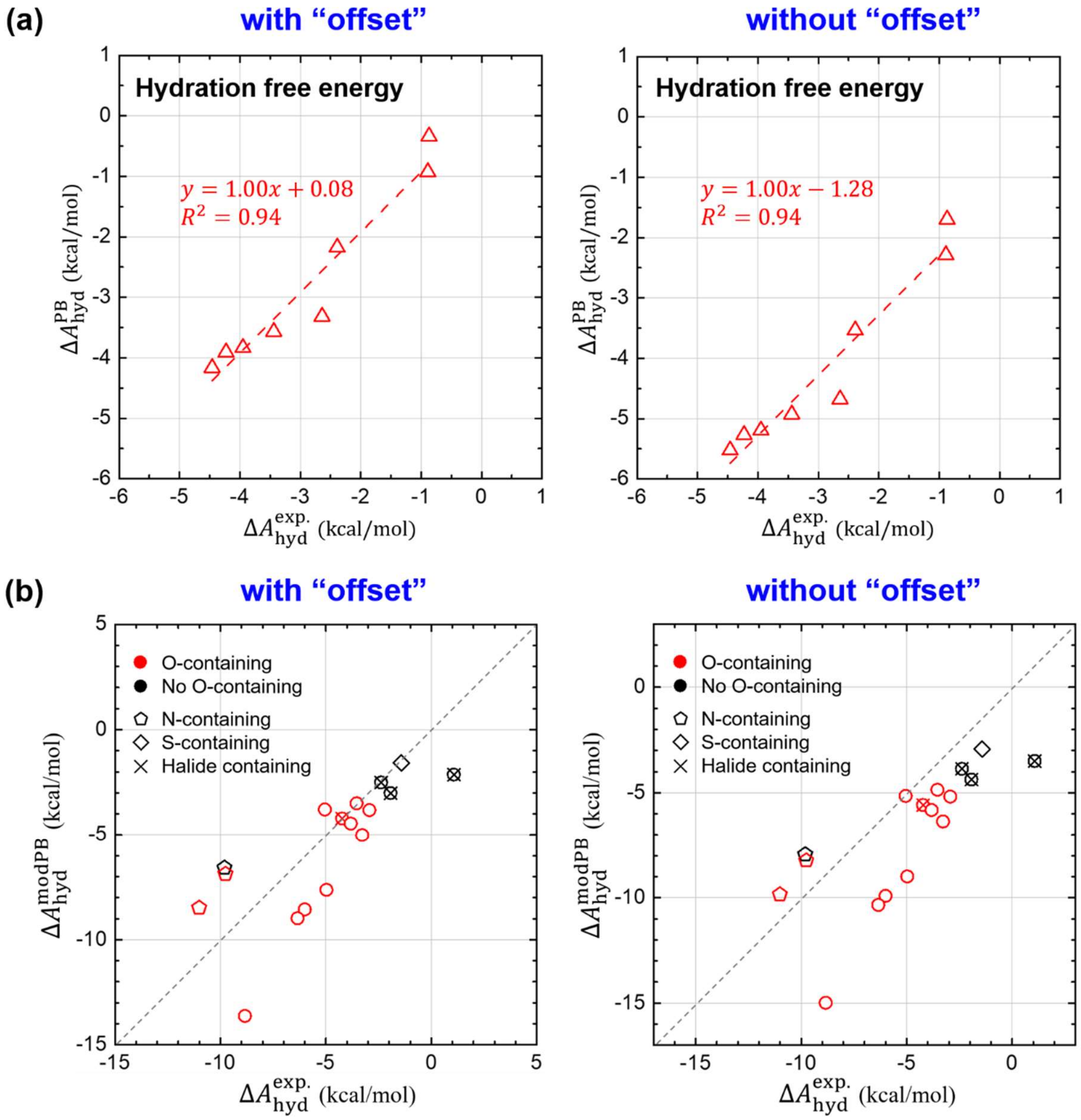

Figure S4. Assessment of the modified PB model by using other aromatic hydrocarbons. The additional test sets contains ten aromatic hydrocarbons: *t*-butylbenzene, ethylbenzene, *p*-xylene, *m*-xylene, *o*-xylene, 1,3-dimethylnaphthalene, 2,6-dimethylnaphthalene, 2,3-dimethylnaphthalene, 1,4-dimethylnaphthalene, acenaphthene. The original PB model still seems problematic as shown in Figure 7c (The slope of the linear fitting curve: 0.38). On the other hand, our modified PB model also predicts well for those non-polar compounds (slope: 1.13).

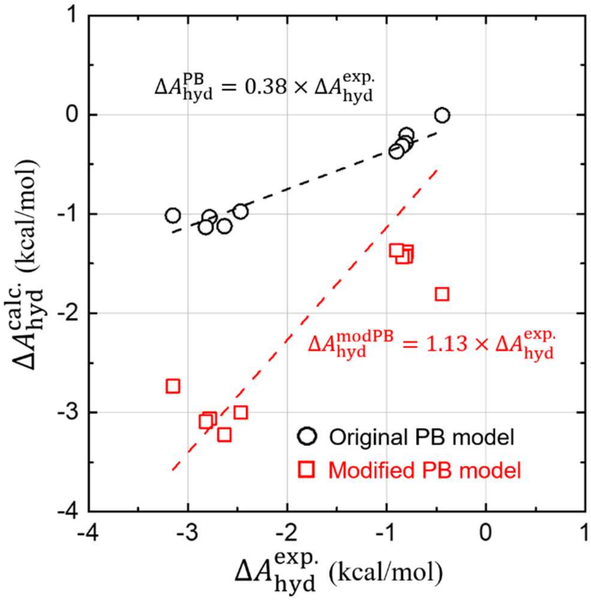

Supplement: Supplementary file 1 [file molecules-23-02927-s001.pdf]
